# Environmental factors influencing the spatio-temporal distribution of *Carybdea marsupialis* (Lineo, 1978, Cubozoa) in South-Western Mediterranean coasts

**DOI:** 10.1371/journal.pone.0181611

**Published:** 2017-07-26

**Authors:** Antonio Canepa, Verónica Fuentes, Mar Bosch-Belmar, Melissa Acevedo, Kilian Toledo-Guedes, Antonio Ortiz, Elia Durá, César Bordehore, Josep-Maria Gili

**Affiliations:** 1 Escuela de Ciencias del Mar, Pontificia Universidad Católica de Valparaíso, Valparaíso, Chile; 2 Marine Biology and Oceanography Department, Institut de Ciències del Mar, Consejo Superior de Investigaciones Científicas, Barcelona, Spain; 3 Dipartimento di Scienze e Tecnologie Biologiche ed Ambientali, Università del Salento, Lecce, Italy; 4 Department of Marine Sciences and Applied Biology, University of Alicante, Alicante, Spain; 5 Departamento de Ecología e Instituto Multidisciplinar para el Estudio del Medio “Ramon Margalef” IMEM, Universidad de Alicante, Alicante, Spain; University of Genova, ITALY

## Abstract

Jellyfish blooms cause important ecological and socio-economic problems. Among jellyfish, cubozoans are infamous for their painful, sometimes deadly, stings and are a major public concern in tropical to subtropical areas; however, there is little information about the possible causes of their outbreaks. After a bloom of the cubomedusa *Carybdea marsupialis* (Carybdeidae) along the coast of Denia (SW Mediterranean, Spain) in 2008 with negative consequences for local tourism, the necessity to understand the ecological restrictions on medusae abundance was evident. Here we use different models (GAM and zero-inflated models) to understand the environmental and human related factors influencing the abundance and distribution of *C*. *marsupialis* along the coast of Denia. Selected variables differed among medusae size classes, showing different environmental restriction associated to the developmental stages of the species. Variables implicated with dispersion (e.g. wind and current) affected mostly small and medium size classes. Sea surface temperature, salinity and proxies of primary production (chl a, phosphates, nitrates) were related to the abundances of small and large size classes, highlighting the roles of springtime salinity changes and increased primary production that may promote and maintain high densities of this species. The increased primary (and secondary) production due to anthropogenic impact is implicated as the factor enabling high numbers of *C*. *marsupialis* to thrive. Recommendations for monitoring blooms of this species along the study area and applicable to Mediterranean Sea include focus effort in coastal waters where productivity have been enriched by anthropogenic activities.

## Introduction

The negative ecological and socio-economic impacts of jellyfish blooms associated with the increase in anthropogenic use of coastal areas are expected to increase [1,2]. Among jellyfish, the Class Cubozoa is infamous for its powerful and even deadly members, which generate major public concern in tropical and subtropical areas [3,4]. The most infamous species is the sea wasp (*Chironex fleckeri*) and several species of carybdeids which are responsible for the dangerous and increasing Irukandji syndrome [5]. In addition, cubozoans have received much scientific attention due to several aspects of their physiology and ecology [6,7], including the complex visual system, swimming and orientation capabilities [8,9] and mating behaviours [10]. On the other hand, the ecology of cubozoans populations is still poorly understood [11] with few papers dealing with quantitative spatial distribution (reviewed in [12].

The Mediterranean Sea has been affected historically with blooms of several conspicuous jellyfish species [13–15], reviewed in [16]. Recently, increases in the abundances and frequency of blooms have been proposed [17–19]. In the Mediterranean Sea, *Carybdea marsupialis* is the only reported species of the Class Cubozoa. This medusa was first reported from the Adriatic as a single preserved specimen in the museum of Vienna by Claus and the scarcity of its records were also highlighted [20]. Later [21] reported a first record for the Adriatic Sea in 1989, reviewed in [22, 23]. Even though the first report is not totally clear, the evidence shows that this species was occasionally recorded in the Adriatic and now it shows a constant presence, appearing in high densities [24]. *C*. *marsupialis* is important because of its painful sting [25,26] and recently has been signalled as the second most important stinging jellyfish on the south coast of Italy, leading to important economical impacts [15] due to the negative impacts on coastal tourism. Similarly, along the coast of Denia (south-west Mediterranean coast) where [27] showed an unusually high density during the summer of 2008, *C*. *marsupialis* stung as many as 185 people d^-1^ and caused major public concern.

In order to establish monitoring programs to further develop risk assessment of jellyfish-human interactions, scientific efforts to understand the environmental and anthropogenic factors influencing the abundance and distribution of stinging coastal cubozoans are greatly needed [12]. Furthermore, studies of spatial patterns of jellyfish species (particularly Class Cubozoa) and the effects of environmental conditions on their distribution is a still growing field [28–30]. Most members of this Class are coastal and the most influential variables are changes in salinity associated with the presence and dynamics of riverine runoff, temperature and currents [27,29,31,32], changes in primary production associated with inter-annual climatic fluctuations [30] and the presence of sandy bottom and/or seagrass (algae) coverage [27,33–35]; for a synthetic analysis see [12].

Beside this, the population dynamics and distribution of cubozoan shows discrete populations units of self maintained genetic diversity [12]. Associated to the physical and oceanographic limitation (currents, topography), natural populations of cubozoans presents highly patchy distributions leading to methodological constrains during the sampling [36] and also resulting in over-dispersion during data analyses [37]. Thus, the analyses of (patchy) count data must be assessed using the proper family distribution, like Poisson or negative binomial, or specifically zero-inflated models [38,39]. The usage of zero-inflated models allows for the identification and effects assessment of 1) “true zeroes”, resulting from real ecological effects like demographic processes, competition, or poor habitat quality (e.g., absence of proper substrate for the polyps), or by the patchy distribution of the species in a three dimensional environment and 2) “false zeroes”, resulting from the incapacity or failing into detect the species (e.g., fast response swimming to net detection) even when the site was suitable [37].

Therefore, the main objectives of the present study were to evaluate the role of environmental and anthropogenic variables on the abundance and spatio-temporal distribution of a coastal cubozoan species, *Carybdea marsupialis*, which has negative interactions with humans and to provide recommendations for the establishment of monitoring programs.

## Materials and methods

### Study site

**Fig 1:**
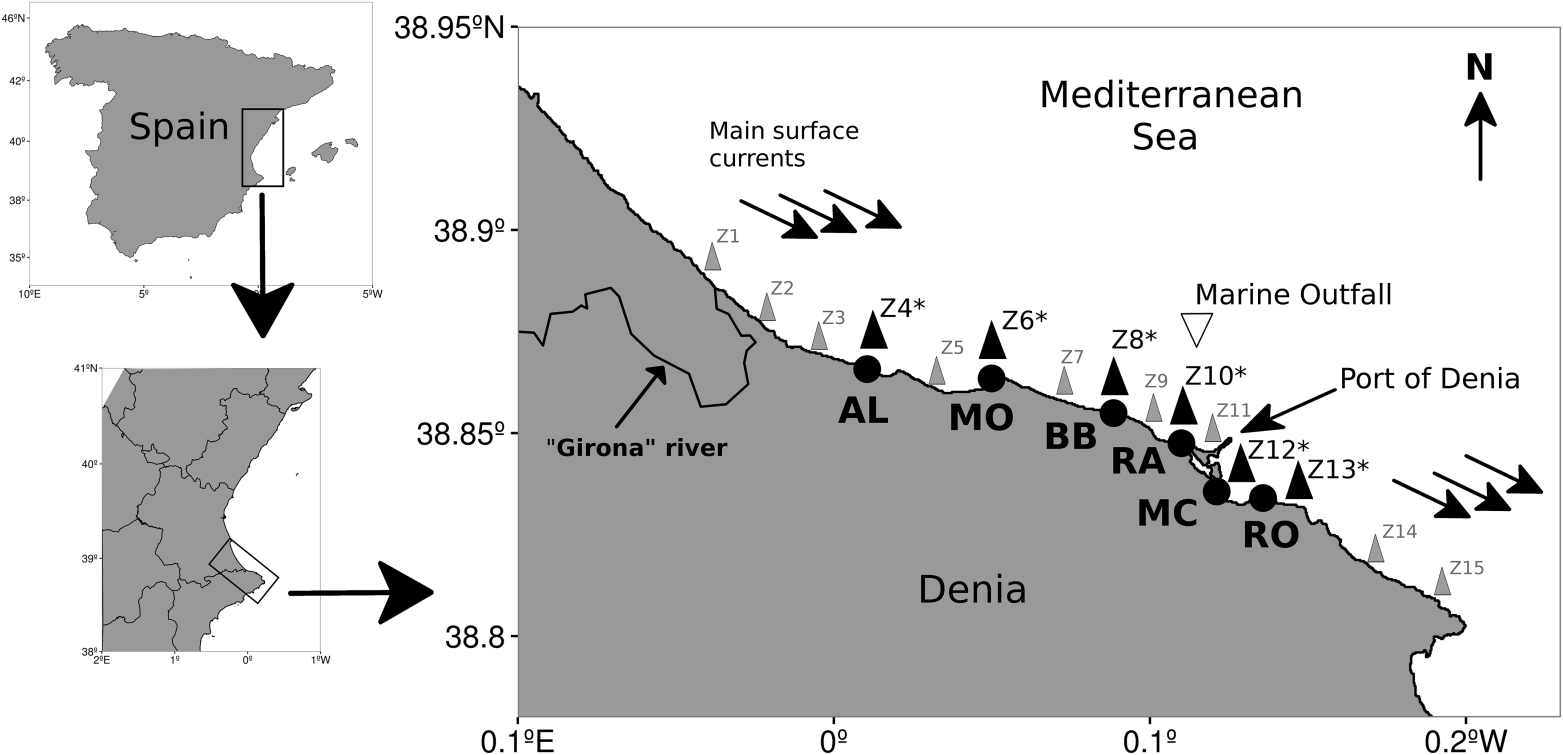
Study area for *Carybdea marsupialis* medusae along the coast of Denia, Spain (Western Mediterranean Sea). Medusae, environmental data and nutrient samples were collected from stations labeled in bold with an asterisk and labeled (Almadrava ‘AL’, Molins ‘MO’, Blay beach ‘BB’, Rasset ‘RA’, Marineta Cassiana ‘MC’ and Les Rotes ‘RO’). The stations marked in gray were not sampled for medusae and are not further considered. The general current flow, the Girona River and the sewage outfall are also indicated.

The study area was located at the south of Valencia Gulf along the coast of Denia (Western Mediterranean Sea). The geographic extension of the study site ranged between 38° 52’ 01.28” and 38° 50’ 03.12” latitude N, covering ~ 12 kilometres. At this area six sites were selected, from North to South: Almadrava ‘AL’, Molins ‘MO’, Blay beach ‘BB’, Rasset ‘RA’, Marineta Cassiana ‘MC’ and Les Rotes ‘RO’ (Fig 1). Beach morphology ranged from sandy beaches with high slopes (AL), sandy beaches with very low profiles and meadows of the algae (*Caulerpa fructifera*) (MC), sandy beaches with rocky reefs and seagrass (*Posidonia oceanica)* meadows (MO and BB) to hard bottom substrate covered mostly by the “photophillic” algae community (RO) [40]. For a full site description see [27].

### *Carybdea marsupialis* medusae sampling

Cubomedusae surveys were conducted from the beginning of July 2010 to the end of July 2011 covering an annual cycle. The abundance of *C*. *marsupialis* medusae was measured weekly at three coastal transects of 20 meters length and a bottom depth between 0.50 to 1.60 m at each of six sites (Fig 1). Three kinds of nets were used on each transect: 200 μm, 500 μm and 4 mm mesh sizes to include all developmental stages of the medusa phase. To estimate the volume of water filtered by each net, a G.O.ENVIROMENTAL (MODEL 2030H) flow meter was deployed in the mouth of each net.

The captured cubomedusae were anaesthetized with menthol crystals [41] for 20 to 40 minutes and then preserved in buffered sea water-formaldehyde solution (4%). The Diagonal Bell Width (DBW) defined as the distance between the bases of two opposite pedalia (which bear the tentacles), was measured with a calibrated stereoscopic microscope (Leica S8APO) for medusae ≤10 mm wide and with Vernier callipers (precision ± 0.05 mm) for larger medusae. Afterwards, medusae were grouped in three categories representing different developmental stages [23] based on the DBW: small (≤ 5 mm), medium (between 5 and 15 mm) and large (≥ 15 mm) individuals.

The Spanish Ministry of the Environment (Dirección General de Sostenibilidad de la Costa y el Mar) and the regional environmental authority (Dirección General del Agua) supported LIFE CUBOMED project and authorised samplings. The field studies did not involve endangered or protected species.

### Environmental variables sampling

Sea surface temperature and salinity profiles were taken in three points at the same time than the transects of medusae abundance. Data was recorded using a CTD (Compact-CTD Lite, model ACTD-CMP; 1 second for reading intervals). Surface currents vectors were obtained by deploying a drift buoy at each beach and recording time and its geographical position by means of a GPS Garmin (GPS 72H), at the beginning and at the end of the three transects. Sampled stations form part of a larger monitoring program along the coast of Denia covering more than 20 kilometres from Z1 (northern station) to Z15 (southern station) (Fig 1). From each spatially coincident stations (marked in bold and with an asterisk in Fig 1), 5 litres of surface water were taken and two replicates from 500 to 1500 ml (depending on turbidity of the water) were filtered. Chlorophyll a (chl a), suspended particulate matter (SPM), phosphate and nitrate data were obtained. For chl a analysis water was filtered through a 47 mm diameter GF/F glass fibre filter. Quantification was measured after a 24 hours extraction with 90% acetone solution in dark and constant temperature (4°C) conditions. The extract (90% acetone was used as a solvent) was transferred into the measuring cuvette of a TURNER fluorometer; and measurements were always done against a blank (reference) cuvette. The fluorometer used a wide excitation band around 450 nm and measured at 670 nm. Then, the extract was acidified and measured again after hydrochloric acid was added, although correction for phaeopigment was not applied in our analysis. Total chl a (μg l^-1^) was then calculated following Wasmund et al. (2006). The average chl a content of each sampling station was used in our analysis. For the Suspended Particulate Matter (SPM) analysis, surface seawater was filtered through a (pre-weighed) 25 mm diameter GF/F glass fibre filter and preserved at -20°C. SPM filters were placed in the stove at 60°C for 24 hours and after this period were weighted using a micro-balance (Mettler Toledo MX5, precision 1 μg). Total SPM (both organic and inorganic) content in the water was calculated as the difference between pre-weighed and dried filter weight, divided by the volume of water filtered for each sample. The average SPM content (μg l^-1^) of each sampling station was used in our analysis. For the nitrate and phosphate analysis, two 10-ml samples of sea water were frozen at -20°C. Nutrient analysis of the samples was performed by the Nutrient Analysis Service at the Marine Science Institute (ICM-CSIC) with an AA3 (Bran + Luebbe) system (formerly known as Technicon), and the mean quantity of nitrate and phosphate (μmol l^-1^) of each sampling station were used. The concentration of nitrate and phosphate are referred to anthropogenic forces in coastal habitats. Wind speed (m s^-1^) and direction (grades) were obtained from a coastal meteorological station at Dènia (http://www.xuss.es).

### Statistical analysis

The abundance (response variable) of the total medusae as well as the three developmental stages (small, medium and large) were analysed. For the modeling process, a subset of the environmental (explanatory) variables was selected in order to avoid collinearity. A Pearson product-moment correlation test was used to elucidate any significant relationship among environmental variables [42]. If the Pearson's correlation coefficient between each pair of the environmental variables tested was higher than 0.5, the least important variable (in terms of its biological-ecological theoretical relationship) was dropped from the model.

Different models were used to describe and characterize the relationship among *Carybdea marsupialis* abundance (counts) and the explanatory (environmental) variables selected. The models used were: Generalized Additive Models using a Poisson (GAM-P) and a negative binomial (GAM-NB) error family distribution and Zero-inflated Models using a Poisson (ZI-P) and a negative binomial (ZI-NB) error family distribution. All the models had a logistic-link function. The error family distribution selected allowed for heterogeneous, discrete (integers) and always non-negative data. Because the abundance (count) of *Carybdea marsupialis* depends on the sample size, all the models used the filtered volume as an offset [43,44]. The general mathematical formulations for the mean, variance, models structure and error distribution used are summarized in S1 Table.

The optimal model was obtained through a backwards selection criteria, based on the significance of each explanatory variable and using the Akaike's Information Criterion (AIC) [45]. This method negatively penalizes excess parameters, preventing from over-parameterization and allowing for multi-model comparison (i.e. the lower the AIC value, the better the model). Models where the same predictors were used, except for one which is set to zero (iteratively), are called nested models (Zuur et al. 2009) and were compared using the likelihood ratio test, using the function *lrtest* from the “lmtest” R package [46].

Finally all the optimal models (GAM-P, GAM-NB, ZI-P and ZI-NB) were visually validated and compared using AIC, log likelihood and degrees of freedom (Df). To assess the degree of concordance between the observed and fitted values, the Pearson correlation coefficient (r) and the Spearman rank correlation (p) were obtained. Additionally, the intercept and slope from a linear regression between the observed versus fitted values were obtained in order to test the hypothetical perfect fit, where the intercept equals 0 and the slope equals 1, as suggested by [44].

All the figures and statistical analyses were done using the statistical platform R, version 3.0.2 (R Core Team 2013). For the GAM models the “mgcv” package [47] and for the zero-inflated models the “pscl” package [48], were used.

## Results

### Spatio-temporal distribution of *Carybdea marsupialis*

**Fig 2:**
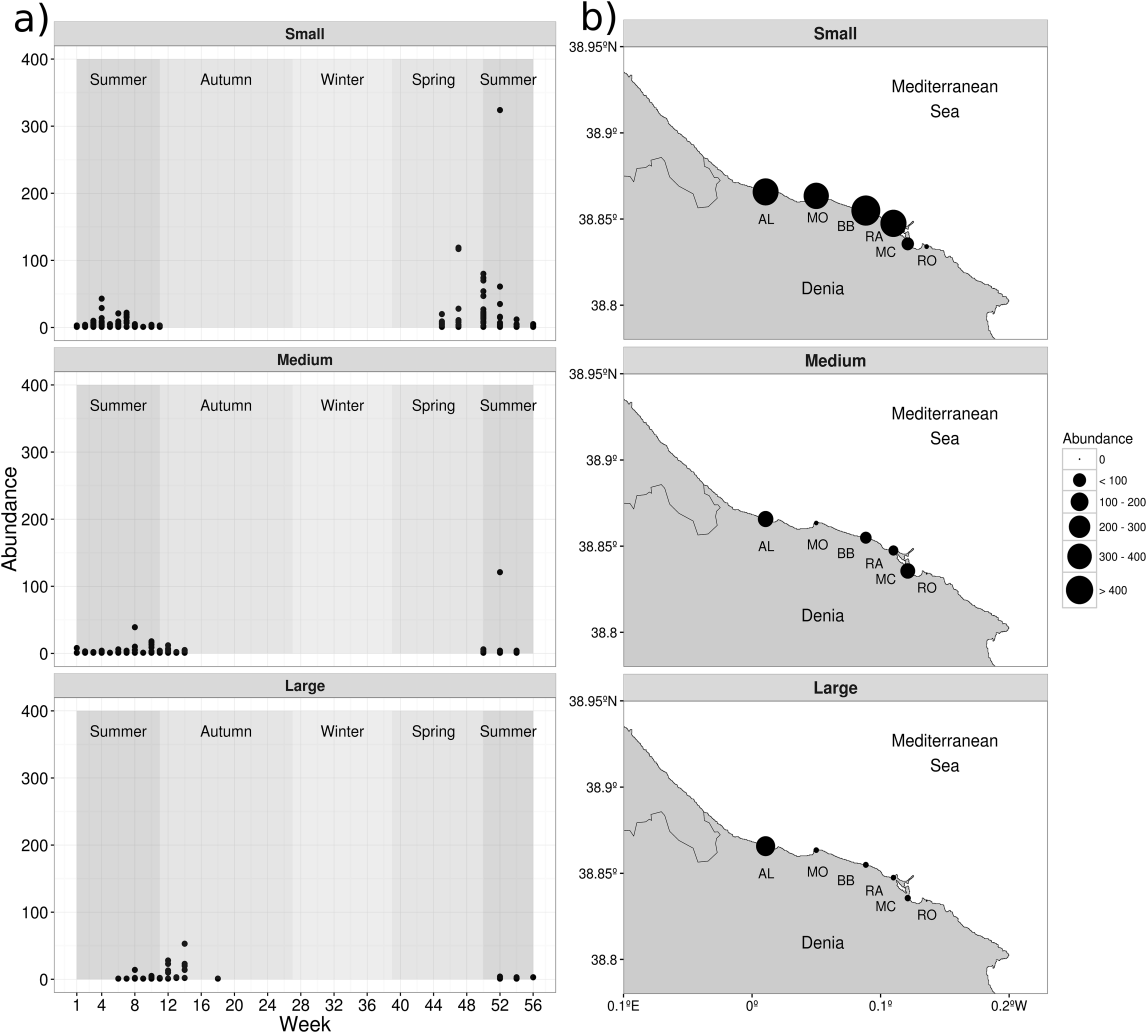
Spatio-temporal variability of *Carybdea marsupialis* medusae collected along the coast of Denia. a) temporal captures from the beginning of July 2010 to the end of July 2011,where shaded areas represent the season period corresponding to the weeks sampled. b) spatial captures at different sites (Almadrava ‘AL’, Molins ‘MO’, Blay beach ‘BB’, Rasset ‘RA’, Marineta Cassiana ‘MC’ and Les Rotes ‘RO’) of Denia. Upper, middle and lower panels show the small, medium and large medusae, respectively.

Weekly numbers of medusae showed high inter-site variability. All sites, except AL, had weeks with no medusae collected, while maximum abundance varied dramatically (Fig 2). The temporal captures showed a succession in size (reflecting growth) of medusae through the sampling period (Fig 2b). The small medusae (DBW = 1.8 ± 1.1 mm) were the first to be caught during spring and until the end of the summer (Fig 2a, upper panel) and the large medusae (DBW = 19.8 ± 4.6 mm) were caught from the summer until mid- Autumn season (Fig 2a, lower pannel). Spatial distribution of captures, showed that small individuals (DBW ≤ 5 mm) were the most abundant and were found relatively equally among beaches, except for MC and RO which accumulated the fewest number of small medusae (89 and 7 individuals, respectively) (Fig 2b). Site AL had high accumulated captures of all medusa sizes small (452), medium (147) and 10-times the number of large medusae than at other sites (Fig 2b; For a summary of the descriptive statistic of the studied population, see S2 Table).

### Spatio-temporal variability of environmental variables

**Fig 3:**
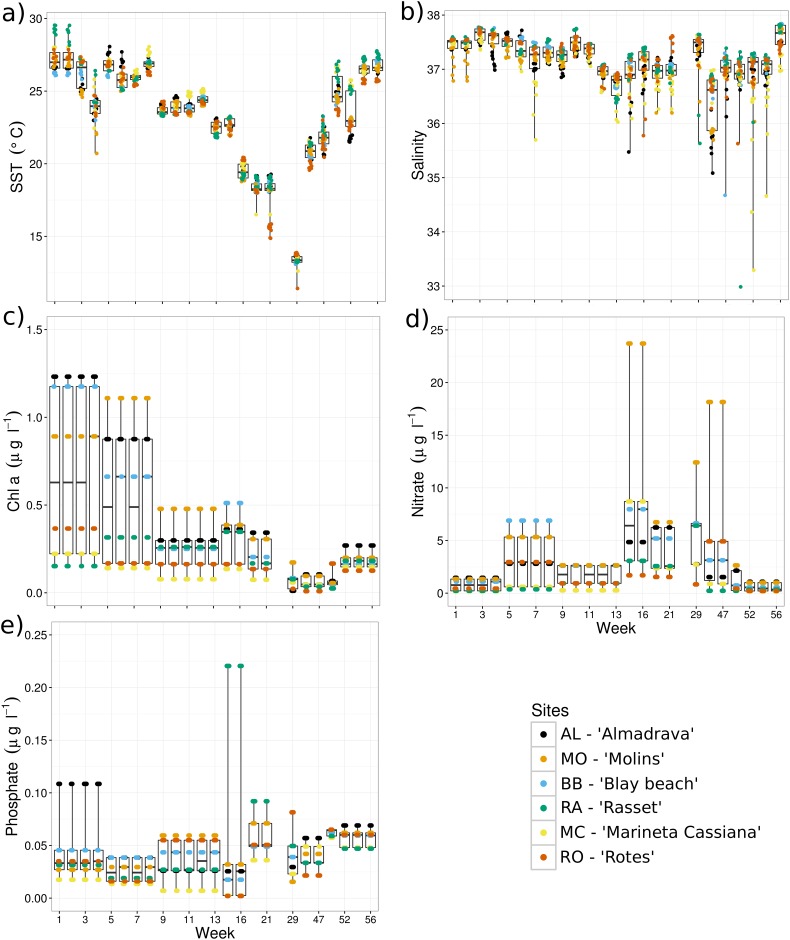
Temporal variability of a) sea surface temperature SST (°C), b) salinity, c) chl a (μg l^-1^), d) nitrate (μmol l^-1^) and e) phosphate (μmol l^-1^), from the beginning of July 2010 to the end of July 2011. The horizontal lines of the whisker-boxplots represent the medians, the boxes represent the interquartile ranges and the whiskers show the ranges of observations. A color-blind friendly legend is used to identify the values from different sampling sites along the coast of Denia, Almadrava ‘AL’, Molins ‘MO’, Blay beach ‘BB’, Rasset ‘RA’, Marineta Cassiana ‘MC’ and Les Rotes ‘RO’.

**Fig 4:**
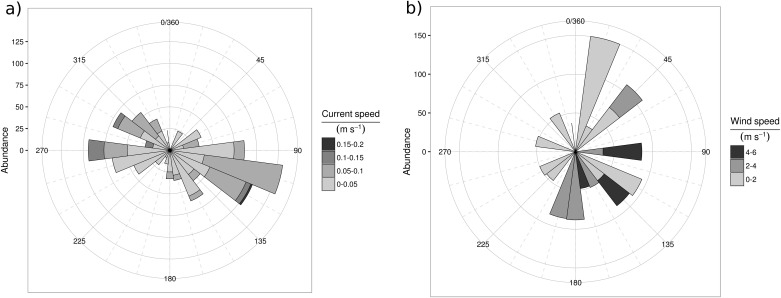
Polar plots showing the a) current direction (degrees) and speed (four categories, m s^-1^) and b) wind direction (degrees) and speed (three categories, m s^-1^) from the beginning of July 2010 to the end of July 2011. Concentric circles represent the abundance (in discrete ranges following the y axis label) of *Carybdea marsupialis* collected. For both polar plots grade range from 0–180 represents the on-shore direction.

Sea surface temperature (SST) typically followed an annual seasonal cycle, with minimum values during the winter (mean ~ 13°C) and maximums during the summer periods (mean ~ 27°C). RA registered the highest temperature values mostly during spring-summer months (Fig 3a). Salinity showed less variation through the sampling period, with maximum values during summer (mean ~ 37.5) and lower values during autumn and spring (mean ~ 36.8 and ~36.9, respectively). RA and RO showed the highest salinity values and MC the minimum values over the study period with records lower than 34 (Fig 3b). Chl a concentration was higheer during summer months (mean ~2.03 μg l-1), especially at the beginning of the sampling period. The lowest values were recorded during winter early-spring months (mean ~ 0.04 μg l^-1^). Additionally, chl a concentration showed a high among-site variability where AL, MO and BB (from north to south) showed the highest concentration during the summer months of 2010 (> 1.5 μg l^-1^). By the contrast, RA, MC and RO (from north to south) showed low values (< 1.0 μg l^-1^) during the whole study period (Fig 3c). Nitrate showed a seasonal cycle with the highest concentrations (average ~ 12 μmol l^-1^) found during winter and spring and the lowest values during the summer period. One site (MO) showed a remarkable peak (23.71 μmol l^-1^) during winter (Fig 3d). Phosphate concentration showed an increase from winter to summer, but it was not common to all sites. Particularly, AL showed a clear peak in summer 2010 (0.11 μmol l^-1^), but RA had an opposite pattern with highest concentrations (0.22 μmol l^-1^) during autumn (Fig 3e). Suspended particulate matter (SPM; not shown) showed high between-site variability with a general increase throughout the sampling period mostly for those sites with soft bottom and/or with low or few seagrass / algae coverage; probably associated to coastal dynamics. Currents flowed mostly parallel to the coast with a general southward direction and high between-site variability, reflecting the effect of beach orientation and coastal morphology (Fig 4a). In general, a positive onshore transport was evident for AL, RA and MC, where SW and W were the main current directions, with flow parallel to the coast at BB and MO, and transport offshore at RO. Sites MO and RO showed the highest current speeds for most of the sampling period (S3 Table). The main wind direction was N—NE with low speed (category 1 = 0–2 m s^-1^), followed by S—SSW winds at medium speed (category 2 = 2–4 m s^-1^) and finally by E—SE winds with the highest velocities (categories 1–3, < 6 m s^-1^) (Fig 4b). Wind speed ranged from 0.75 (m s^-1^) during autumn to 5.40 (m s^-1^) during the summer months with the lowest variability values recorded during the winter months (S3 Table).

### Statistical analysis

#### Variable selection

The Pearson’s correlation coefficient was lower than 0.5, except for the correlation between SPM and nitrate (r = 0.52, p-value <0.01). In this case, SPM was dropped from the models (S1 Fig).

#### Model results

**Fig 5:**
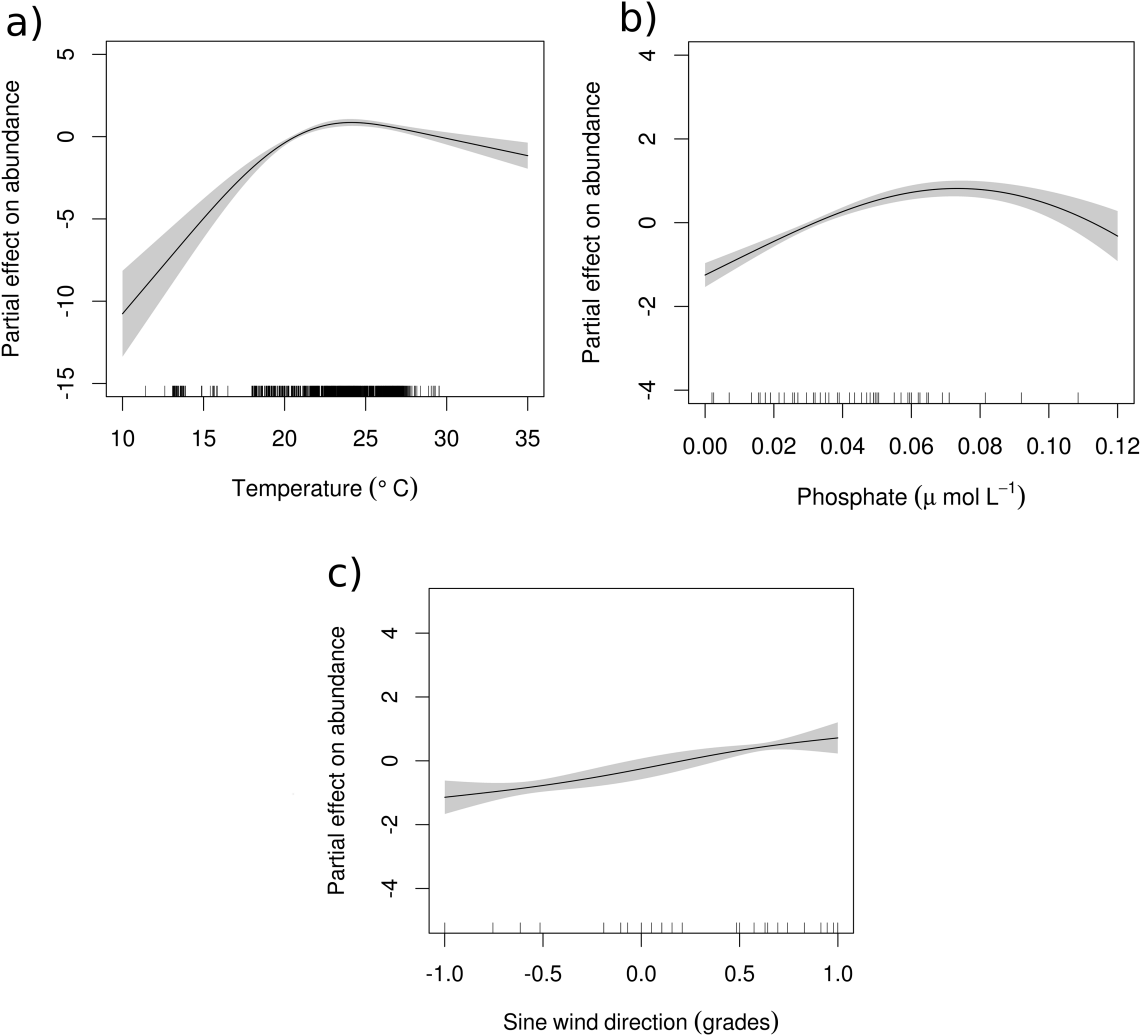
Partial effects of a) temperature (°C), b) phosphate (μ mol L^-1^) and c) wind direction (sine degrees), over the abundance of total *Carybdea marsupialis* medusae collected along the coast of Denia. Central (bold) line show the best fit and the shaded areas show the 95% confidence intervals of the GAM-NB model. Bottom vertical lines represent observations.

Models based on the negative binomial distribution performed better than the all other tested models (S4 Table). Thus, interpretation of the results were based on GAM-NB and ZI-NB models. Concordance between those models, showed that total captures were modelled by temperature, phosphate and wind direction (sine) and from the ZI-NB model, temperature and wind speed were significantly affecting the number of false zeros (zero-inflation; Table 1). The partial effect of temperature showed a positive relationship with medusae abundance until a maximum of ~ 25°C, after that temperature had a negative effect (Fig 5a). Phosphate showed a similar positive relationship until a maximum at ~ 0.07 μmol l^-1^ (Fig 5b). Different size classes were affected differently by the environmental variables. Parametrization of the optimal models (those with all the explanatory variables been significant) for the three size class are shown in Table 2. Small size class was affected mostly by variables associated with transport (wind and current direction and intensity) and productivity (phosphate and chl a). The zero inflation was explained mostly by the effect of wind, where strong Easterly winds and offshore transportation (i.e. negative values of the wind sine direction slope) lead to higher than expected, by the environmental condition, number of zeros. GAM-NB model explained a total of 60.0% of the deviance. Temperature had a positive effect on the abundance of small medusae with a maximum at ~23°C where further increases in temperature did not affect their abundance (S2a Fig). Nitrate and phosphate also showed an unimodal response, where positive effect on small medusae abundance was achieved at the optima (~ 7 μmol l^-1^ and 0.09 μmol l^-1^ for nitrate and phosphate, respectively), afterwards the general effect was negative (S2a Fig). Medium class was mostly affected by temperature and by dispersive variables like wind (speed and direction) and current speed. Because of no zero-inflation, ZI-NB models were not fitted to medium size class. The GAM-NB model was able to explain 51.1% of the deviance. Temperature had a significant unimodal response (similar to small class), with an optimum value ~ 26°C. However, the negative effect over ~ 27°C showed higher uncertainty (S2b Fig). A similar pattern was found for wind speed where values until ~ 3.5 m s^-1^ had a positive effect on abundance, afterwards the abundance was reduced (S2b Fig). Medusae abundance was affected positively by westerly winds and by the current speed (Table 2). Large class was affected positively by temperature and by variables indicatives of productivity (nitrate, chl a and phosphate). Also wind and current played an important role in their abundance, but opposite to small class, no effect was detected for these variables over the zero-inflation. In this case temperature and chl a were the responsible variables for the higher than expected number of zeros (Table 2). The explained deviance for the GAM-NB was 72.4%. Temperature showed a positive pattern on the abundance of large medusae with a maximum effect at ~ 23°C (S2c Fig). Current speed showed a non-linear pattern where a negative effect was evident until velocities near ~0.8 m s^-1^, afterwards the increase in current speed showed a positive effect (although few observations and wide confidence intervals; S2c Fig). Predominant currents towards the south tended to diminish the abundance of large medusae but its effect was site specific due to the geographical orientation of the sampling area.

**Table 1:** Model results for total *Carybdea marsupialis* medusae collected on the coast of Denia. The selected environmental variables and values for Akaike's information Criterion (AIC), Log likelihood (Log lik) and degrees of freedom (Df) of the models are shown.

Model	Parameter	Slope	z-value	p-value	AIC	Log lik	Df
ZI-NB (Count model coefficients)	Temperature	-0.13	-2.5	< 0.05			
	Phosphate	23.52	3.24	< 0.001			
	Sine (wind direction)	0.98	3.62	< 0.001	2507.8	-1246	8
ZI-NB (ZI model coefficients)	Temperature	-0.68	-4.63	< 0.001			
	Wind speed	-2.42	-3.13	< 0.05	2507.8	-1246	8
GAM-NB	Temperature	1.98	69.1	< 0.001			
	Salinity	2.96	29.63	< 0.001			
	Wind speed	1.99	180.35	< 0.001			
	Sine (wind direction)	1.68	74.65	< 0.001			
	Cos (wind direction)	2.92	166.5	< 0.001			
	Current speed	2.85	15.37	< 0.01			
	Sine (current direction)	2.91	40.38	< 0.001			
	Chl a	2.92	47.23	< 0.001			
	Nitrate	2.94	46.94	< 0.001			
	Phosphate	1.97	79.82	< 0.001	3743.4	-1845.6	26

**Table 2:** Model results for small, medium and large *Carybdea marsupialis* medusae collected along the coast of Denia. The selected environmental variables and values for Akaike Information Criterion (AIC), Log likelihood (Log lik) and degrees of freedom (Df) of the models are shown. (in case of “medium size cubozoans” The ZI-NB models were not fitted because the distribution was not zero-inflated).

Model “small size”	Parameter	Slope	z-value	p-value	AIC	Log lik	Df
ZI-NB (Count model coefficients)	Temperature	-0.22	-4.2	< 0.001			
	Nitrate	-0.14	-3.7	< 0.001			
	Phosphate	23.3	3.6	< 0.001	1646.9	-798.5	25
ZI-NB (ZI model coefficients)	Temperature	-0.4	-2.8	< 0.001			
	Wind speed	-3.6	-3.9	< 0.001			
	Sine (wind direction)	-2.5	-3.9	< 0.001			
	Chl a	-5.3	-3.3	< 0.001	1566.9	-773.4	10
GAM-NB	Temperature	1.95	26.63	< 0.001			
	Salinity	2.96	36.21	< 0.001			
	Wind speed	2.00	70.90	< 0.001			
	Sine (wind direction)	3.00	89.62	< 0.001			
	Cos (wind direction)	2.95	52.92	< 0.001			
	Sine (current direction)	2.99	31.96	< 0.001			
	Cos (current direction)	2.91	21.38	< 0.001			
	Chl a	2.96	66.55	< 0.001			
	Nitrate	2.15	26.85	< 0.001			
	Phosphate	2.00	67.47	< 0.001	1663.6	-808.5	23
Model “medium size” GAM-NB	Parameter	Slope	Chi-squared	p-value	AIC	Log lik	Df
	Temperature	1.89	17.20	< 0.001			
	Wind speed	1.98	38.84	< 0.001			
	Sine (wind direction)	1.00	11.59	< 0.001			
	Current speed	2.91	24.47	< 0.001	473.6	-228	9
Model “large size” ZI-NB (Count model coefficients)	Parameter	Slope	z-value	p-value	AIC	Log lik	Df
	Temperature	-0.49	-3.8	< 0.001			
	Current speed	-25.27	-4.37	< 0.01			
	Cos (Current direction)	-1.54	-4.52	< 0.001			
	Nitrate	-0.3	-2.33	< 0.05	490.4	-220.2	25
ZI-NB (ZI model coefficients)	Temperature	-1.5	-3.72	< 0.001			
	Chl a	-39.81	-3.26	< 0.001	487.6	-234.8	9
GAM-NB	Temperature	1.92	12.81	< 0.01			
	Wind speed	1.82	24.69	< 0.001			
	Sine (wind direction)	2.19	8.22	< 0.05			
	Cos (wind direction)	2.99	9.68	< 0.05			
	Current speed	2.70	77.93	< 0.001			
	Cos (current direction)	2.34	20.19	< 0.001			
	Chl a	2.95	54.54	< 0.001			
	Phosphate	2.00	27.61	< 0.001	474.9	-217.6	20

## Discussion

### *Carybdea marsupialis* medusae

Studies dealing with abundance and spatio-temporal distributions of cubozoans remain scarce (Bentlage et al. 2009, Bordehore et al. 2011, Chiaverano 2013, For a synthetic analysis see Kingsford & Mooney 2014). Here we studied the spatio-temporal dynamics and association with environmental variables of a local population of the cubozoan *Carybdea marsupialis*. In general, those beaches with a sandy-bottom associated with patches of seagrass/algae showed the highest abundances and those with hard bottom and photophilic algae community showed the lowest abundance values. In a study conducted during 2008 and 2009 in the same area, [27] using a hand net of 5-mm mesh (comparable to large and medium medusae abundance in our study) showed lower abundance of cubozoans. At the same site (AL) the maximum density in our study was more than four times greater (53 vs. 18 indiv. m^-3^). In total, for the coast of Denia the mean density of *C*. *marsupialis* medusae in 2010–2011 was twice that in 2008–2009 (0.8 vs. 0.4 indiv. m^-3^) with the average maximum densities been three times greater (16.2 vs. 5.6 indiv. m^-3^). Moreover, Bordehore et al. (2011) did not capture *C*. *marsupialis* medusae on MC and RO Sites (Fig 1) and in our study, the highest mean medusae density was found in MC (2.4 indiv. m^-3^) and again very low captures were recorded at RO site. The fact of find *C*. *marsupialis* south of the port of Denia, highlight the ability of small (recently detached) medusae to drift over medium distance and even to pass human structures and to thrive in places where good conditions are present (underwater visual inspection on the port of Denia structures have not revealed yet the presence of cubozoan polyps). Thus, while MC is a sandy beach with high cover (> 80%) of the green algae *Caulerpa prolifera* and RO site is a rocky-bottom beach fully covered with a benthic photophilic algae community, the establishment of local populations will depend ultimately on the ecosystem characteristics, where for *C*. *marsupialis* the need for sandy bottom with seagrass/algae meadows (absent in RO site) seems evident, as has been demonstrated for other cubozoan species, such as *C*. *rastonii*, *Chiropsella bronzie* and *Chiropsoides buitendijki* [33–35], reviewed in [12].

Large *Carybdea marsupialis* (≥15 mm DBW, gonadal tissue becomes visible) are also important from a coastal zone management perspective, because their stings are painful and have become a sanitary problem that can be detrimental for local tourism [15,25,27]. The distribution of large *C*. *marsupialis* medusae was restricted mainly to one site (AL) where 234 medusae (of 285 total) were captured. This site is a sandy beach with *Posidonia oceanica* meadows, high slope and strong coastal dynamics and closely to a river discharge (Girona River) [27]. This site had the highest values of chl a and phosphate, mostly at the beginning of the study period, reflecting the association of large medusae with variables associated to high levels of prey. From an stomach content analysis of juvenile and adults of *C*. *marsupialis* individuals mostly from this site, [49] found that crustaceans was the main prey item, followed by polychaetes and in a lower extent fish larvae. Field observations revealed that high turbidity in the surf zone was related to captures of the largest medusae. One explanation could be their avoidance of high levels of solar irradiance. Photosensitive behaviour and obstacle avoidance is common in cubozoan species [12]. Also, these areas of low visibility and high dynamics could make more of their large prey available and also allow large medusae to remain undetected by their large epibenthic invertebrate prey [49], thereby generating advantageous feeding conditions for these entangling predators [50]. Cubozoans are strong swimmers that can cover distances up to kilometres d^-1^ [8] with complex visual systems [6] that have the ability to select areas with optimum conditions. Thus the high numbers of large medusae associated with soft bottom and patchy meadows of seagrass, as previously reported by [27], can be explained by the abundance of their prey typically found in those ecosystems [51].

### Associations of environmental variables

The effects of environmental variables on the local (~ tens of kilometres) abundance of the cubomedusa *Carybdea marsupialis* showed that temperature was the main factor affecting the spatio-temporal abundance along the SW Mediterranean coast. The temporal variation of sea temperatures reflected the common seasonality found along the Mediterranean Sea [52] and its variability was consistent among sites, with the highest values reported (~ 30°C) showing the effect of the coastal morphology and low beach dynamics found in the study area. From the cubozoan perspective, a sudden increase in temperature after the winter was associated with the appearance of small medusae in the study area, reflecting the role of this parameter in the life cycle of metagenetic species (reviewed in [53,54]).

Other variables that significantly explained the cubozoan abundance were conditions of low salinity and high coastal productivity (chl a and phosphate) when the physical conditions (wind current speed and direction) lead to medusae being recorded at high numbers. Salinity showed low between-site variability and the seasonal dynamics showed the effects of high rain and river discharge during the spring months where the minimum salinity values were recorded. The reduction in salinity was explained by the proximity of the “Girona” River in the northern part of the study area (AL) and by the groundwater discharges in the southern area (MC, RA and RO). Maximum values were recorded in summer and winter associated with dry conditions in the study area [55]. Most small medusae collected were recently detached medusae, possibly suggesting when and where the medusae were produced from the benthic phase (cubopolyps) and the stimuli for metamorphosis. Nevertheless, metamorphosis of *C*. *marsupialis* polyps into medusae along the coast of Denia seemed not to be one synchronized event, because small medusae were sampled from mid-spring to late summer months (Fig 2a upper panel). Temperature and salinity showed sudden changes (increases and decreases, respectively) during the spring period (week 47, Fig 3a y 3b). Concurrently, small medusae were captured in samples in spring 2011 (week 45) (Fig 2a upper panel), suggesting a relationship between changing environmental variables and the metamorphosis of *C*. *marsupialis* cubopolyps. [32] showed that salinity reduction accelerated the metamorphosis of its congener *Carybdea sp*. from Puerto Rico, suggesting an association between the salinity reduction with high rain conditions, resulting in an increase in primary and secondary production [56,57]. Similarly, association of juvenile medusae with estuarine habitats was proposed for the cubozoan *Chironex fleckeri* ([58] cited in [12], [31]), but see [59].

In addition, the abundance of small *C*. *marsupialis* medusae was significantly correlated with chl a, nitrate and phosphate along the coast of Denia. There, fresh water runoff is high but restricted to spring [60] and the surrounding sea is characterized by oligotrophic waters [52,61]. Thus, reduction of salinity with associated increases in nutrients and coastal production increased the production of *C*. *marsupialis* medusae. Chlorophyll a was highest in the three northern sites (AL, MO, BB, from north to south) and lowest values in the three southern sites (RA, MC, RO), with a seasonality showing high mean values (0.76 ± 0.59 μg l^-1^) during summer and low values (0.17 ± 0.11 μg l^-1^) in early spring, in concordance with those reported for the Catalan Sea [62]. Similar positive association with coastal productivity (primary and secondary production) was established from a long-term study in Hawaii, where the cubozoan *Alantina moseri* also was positively associated with zooplankton biomass [30].

The effects of river discharges on the primary production explained the high chl a concentration recorded along the northern sites, as proposed by [55] who found maximum values (11.71 μg l^-1^) associated with large discharge of nutrient-rich waters in an enclosed estuarine area. Even though, northern sites can be influenced by the Girona River, where the temporarily high values of chl a recorded at the beginning of the study period seemed to be an unusual situation. Synergistic effects of fresh water discharge and processes relative to beach nourishment [63] and locally nutrient rich water discharges seemed to occur [27], but we cannot directly address this issue. We speculate that the local industry adds high nitrate loads in freshwater as commonly found in Spain, where there is traditional farming and agricultural industry [64]. Along the coast of Denia, fertilizers are overused in intensive agricultural activities and the excess seeps into ground water, rivers, and streams, letting nitrogen reach the coastal areas in high concentrations [55]. High values of phosphate in coastal areas also are associated with agricultural activities, as well as with the discharge of waste waters from the coastal sewage plants in Denia [55,64]. In this way, increased primary production is associated with high levels of phosphates along the coast of Denia and related to the rainy season in spring when terrestrial runoff and river discharges are enhanced, and also during summer when local tourism increases discharge from the coastal sewage plants (Fig 1, [55,64,65]). Small and large *C*. *marsupialis* medusae were associated with variables related to fresh water discharge (salinity reduction) and/or to local increases in productivity, as indicated by nitrate, phosphate and chl a.

### Zero-Inflation in total captures

The high numbers of zeroes for the total data were explained by just two variables, temperature and wind speed (Table 1). Those variables represent the particular conditions where the amount of zeroes were higher than expected by theory. In the case of wind speed the response is more or less clear, as the behavioural avoidance responses of this species can explain the fact that many transects resulted in zero-inflated captures. Along the coast of Denia, one hypothesis is that medusae are able to select the depth and closeness to the shoreline, as previously demonstrated for large *Chironex fleckeri* medusae, which swam to the calm leeward side of Magnetic Island (North Queensland, Australia) when conditions were choppy on the windward side [58]. Because of the coastal restricted sampling we cannot discuss the distribution of *C*. *marsupialis* further from the coast or vertically through the water column.

Temperature, on the other hand, had a confounding effect because the most captures were of small medusae, which due to growth appeared negatively related to high temperatures (inflating the number of zeroes) as they became medium and large size class. The effect of ontogenetic development led to zero-inflated data for small medusae. The between-site variability in the temperature effect was evident at RA and MC sites, where medusae appeared at the same time and larger than at the other sites; the proportion of medium medusae also occurred earlier at this site than the others (Acevedo unpublished data). One characteristic of these sites is their low profile (slope < 5°) and very shallow depth (0 to 1.5 m) that generate local conditions of high SST in spring, which could have enhanced the growth of small medusae into medium size, resulting in a numerous zeroes for small medusae compared to others sites (S2 Table). Medium size medusae were the most numerous with few zero captures (S2 Table). Their “optimum” response to temperature possibly reflected the fact that at the beginning of the summer, warmer temperatures were related to their growth (environmental conditions support their physiological requirements) and, later, the apparent negative effect of temperature may be due to their transformation into the large size class (ontogenetic growth associated with the investment in reproduction) (S2c Fig).

### Dispersion related variables

The wind speeds (0.75 to 5.40 m s^-1^) and seasonal patterns (S3 Table) were in agreement with those characterized for the coast of Alicante [66], with the main direction (E, SE) characteristic of the sea breeze in this area [67]. During our study, no high values of wind were detected, in part because the sampling was restricted to low to moderate wind speeds (< Beaufort Scale “3” equivalent). Sea surface currents highlighted the predominantly southward current [61] in the NW Mediterranean Sea and also the potential effect of coast morphology, especially the effect of breakwaters on currents that may increase the retention of near shore waters [27]. Dispersion related variables (i.e. wind and current speed and direction) affected mainly the small and medium size classes defined in this study in agreement with their role as the dispersive phase of this species. This reflects that small size medusae behave as passive drifters and may explain the lack of differences in abundance among sampling sites (Fig 2b, upper panel). Variables associated with the zero-inflated data (Table 1) reflect the fact that at high wind stress (rough conditions caused by high wind speeds associated with the effects of beach orientation), the captures of small medusae were zero even when the other variables where considered “good” by the model. The medusa behaviour of avoidance of rough conditions and off-shore transport of small medusae due to wind-driven surface currents may be responsible for the increased number of “false” zeroes. Thus, future sampling for this small and medium size classes should avoid rough sea surface conditions to avoid zero-inflated data.

### Statistical modeling

In general, the models based on the negative binomial error distribution performed better than those with the Poisson based error distribution. For small medusae, the correlation between the observed and the fitted values was higher with GAM-NB model (r and p value = 0.44 and 0.68, respectively), which also had the second lowest AIC (1818.62), followed by ZI-NB (AIC = 1646.90, S4 Table). For the medium size medusae, although GAM-P had a better Pearson correlation (r = 0.60) between the observed and fitted values, the GAM-NB had a much lower AIC value, which indicates a better model. Finally for large medusae, GAM-NB models performed better in all the statistics evaluated, with high Pearson (r) and Spearman rank (p) correlations and with an intercept close to zero (0.1) and slope close to 1 (0.8), which are indicative of a perfect fit [45]. This can be attributed to the fact that large medusae were more restricted in space and time, with less variability in the environmental variables; therefore, the models could explain in greater detail (> 70% of the deviance explained) and predict their abundances more accurately. The poor performance of Poisson error distribution models reflect the fact that count data coming from patchily distributed organisms like gelatinous zooplankton tend to show over dispersion [39] and use of negative binomial error distribution family or zero-inflated models is necessary to account for this characteristic [39,68]. In our study, GAM-NB models had higher correlations between observed versus fitted values, making their predictions of the effects of the environmental variables on the abundance of *C*. *marsupialis* believable. The reason to include the ZI models is based on the fact that these models allowed for ecological interpretation of the underlying processes producing the false zeroes in the medusae counts, resulting in improvement of the ecological analyses [48,68]. In the case of *C*. *marsupialis*, which can congregate at high densities (reviewed in [24]) and its sting causes public concern and important economic costs [15], it is important to accurately assess the risk and to develop monitoring programs. Tools that promote understanding which variables explain its distribution, and also conditions that will maximize the efficiency of the survey effort are of high value [12]. Thus, our work presents important and highly needed information about seasonality, spatial patterns and the influence of environmental factors on the abundance of a coastal box jellyfish and also recommendations for the establishment of monitoring programs.

## Conclusions and recommendations for monitoring and mitigation

The distribution of *Carybdea marsupialis* medusae seems to reflect the dispersion of the small and medium size classes by the onshore transport due to surface currents and high levels of primary and secondary production associated with the bottom type (sandy bottom and algae/seagrass meadows). Those factors allow *C*. *marsupialis* medusae to thrive where high densities were also recorded previously [27]. *C*. *marsupialis* have been recorded several places in the Mediterranean Sea, but rarely generating blooms (sensu [69], reviewed in [24]). Along the coast of Denia, high abundances of *C*. *marsupialis* were associated with variables indicating high local productivity. In those areas, coastal production is based on the discharge of rivers that deliver not only fresh water and terrestrial sediment to the coast, but also large amounts of nitrate and other crop fertilizers [27,55], enhancing the coastal productivity in this area [64]. Particularly in AL, in addition to the combination of high levels of chl a and phosphate, [27] suggested the role of stone breakwaters as new substrata that enhanced the settlement of the benthic stage of this species. Similarly, along the Italian Adriatic shore stinging reports of *C*. *marsupialis* have increased in recent years [15], as well as installations of several stone breakwaters parallel to the coast (F. Boero, personal communication). Thus, the blooming of *C*. *marsupialis* medusae associated with coastal areas can be a warning of coastal eutrophication and general environmental degradation.

Taken together these findings we suggest that future studies should consider the following recommendations. First, in order to detect the presence and estimate the abundance of *C*. *marsupialis*, coastal sampling should start in late-winter (just before the temperature start to increase) and use ~ 500 μm mesh size to collect the small medusae. Sampling during rough sea conditions should be avoided to minimize underestimation and false negative records. Once the population is established and the presence of medium size is confirmed, the large medusae should be sampled with greater mesh size (~ 4 mm) to collect the most, large stinging individuals. In this case, sampling should be restricted to popular beaches with sandy bottom associated with three dimensional structures like seagrass/algae meadows, new breakwaters or other artificial substrata. Similarly, for beaches with low wave dynamics and higher temperature due to shallow profiles, sampling for large individuals should start earlier in those sites. Second, crop fertilizers and sewage discharges should be reduced in highly populated or industrialized areas. Most importantly, high amounts of nutrients reaching the coast (by rivers or ground water discharges) should be prevented, especially in early spring. In order to decrease the amount of sewage water, solutions are related with the improvement in technology adding the tertiary biological treatment to the sewage plants and by relocating the submarine pipeline farther away to the coast, in places where the currents will spread the effluents. The idea behind this recommendation is to diminish the secondary production mostly in those places where habitat is good for *C*. *marsupialis*, soft bottom, sandy to gravel-sandy beaches and seagrasses meadows.

## Supporting information

S1 Fig Collinearity analysis for all environmental variables. Lower-left panels show scatter plots with a loess smoothing line (in red) to help visualize patterns. Central (Diagonal) panels show histograms of the environmental variables and upper-right panels show the values of the Pearson’s correlation coefficients for each pair of variables, with text size proportional to its value.
(TIFF)

S2 Fig Optimal models showing the partial effect on abundance of the significant environmental variables for a) small, b) medium and c) large *Carybdea marsupialis* medusae collected along the coast of Denia. Central (bold) line show the best fit and the shaded areas show the 95% confidence intervals of the GAM-NB model. Bottom vertical lines represent observations.
(TIFF)

S1 Table Mathematical model formulation for the generalized additive models with Poisson family distribution (GAM-P), with negative binomial family distribution (GAM-NB), for the zero-inflated Poisson (ZI-P) and zero-inflated negative binomial (ZI-NB) models (source Zuur et al. 2009). (DOC)

S2 Table Descriptive statistics for the number and density (in parentheses; indiv. m^-3^) of three sizes (Small, Medium, Large) of *Carybdea marsupialis* medusae captured by site (Almadrava ‘AL’, Molins ‘MO’, Blay beach ‘BB’, Rasset ‘RA’, Marineta Cassiana ‘MC’ and Les Rotes ‘RO’) along the coast of Denia. Minimum 'Min', maximum 'Max', sum 'Sum', mean 'Mean' and standard deviation 'SD' values are showed for each class size and sampling site.
(DOC)

S3 Table Descriptive statistics for the selected environmental variables from the beginning of July 2010 to the end of July 2011for all sites surveyed (Almadrava ‘AL’, Molins ‘MO’, Blay beach ‘BB’, Rasset ‘RA’, Marineta Cassiana ‘MC’ and Les Rotes ‘RO’) on the coast of Denia, Spain. Values are shown by season.
(DOC)

S4 Table Model comparison for the generalized additive models with a Poisson family distribution (GAM-P), generalized additive models with a negative binomial family distribution (GAM-NB), zero-inflated Poisson (ZI-P) and zero-inflated negative binomial (ZI-NB) models. The Pearson correlation coefficient (r), Spearman rank correlation (p), intercept (α) and slope (b) of a linear regression between the observed versus fitted values, Akaike Information Criterion (AIC), log likelihood (Log lik) and degrees of freedom (Df) are showed for each size class of *Carybdea marsupialis* medusae. NA = Not Available.
(DOC)
