# Supplementary figures and images for: Environmental factors influencing the spatio-temporal distribution of Carybdea marsupialis (Lineo, 1978, Cubozoa) in South-Western Mediterranean coasts
Source: PLoS One. 2017 Jul 26;12(7):e0181611. doi: 10.1371/journal.pone.0181611 (PMC5528890; doi:10.1371/journal.pone.0181611)

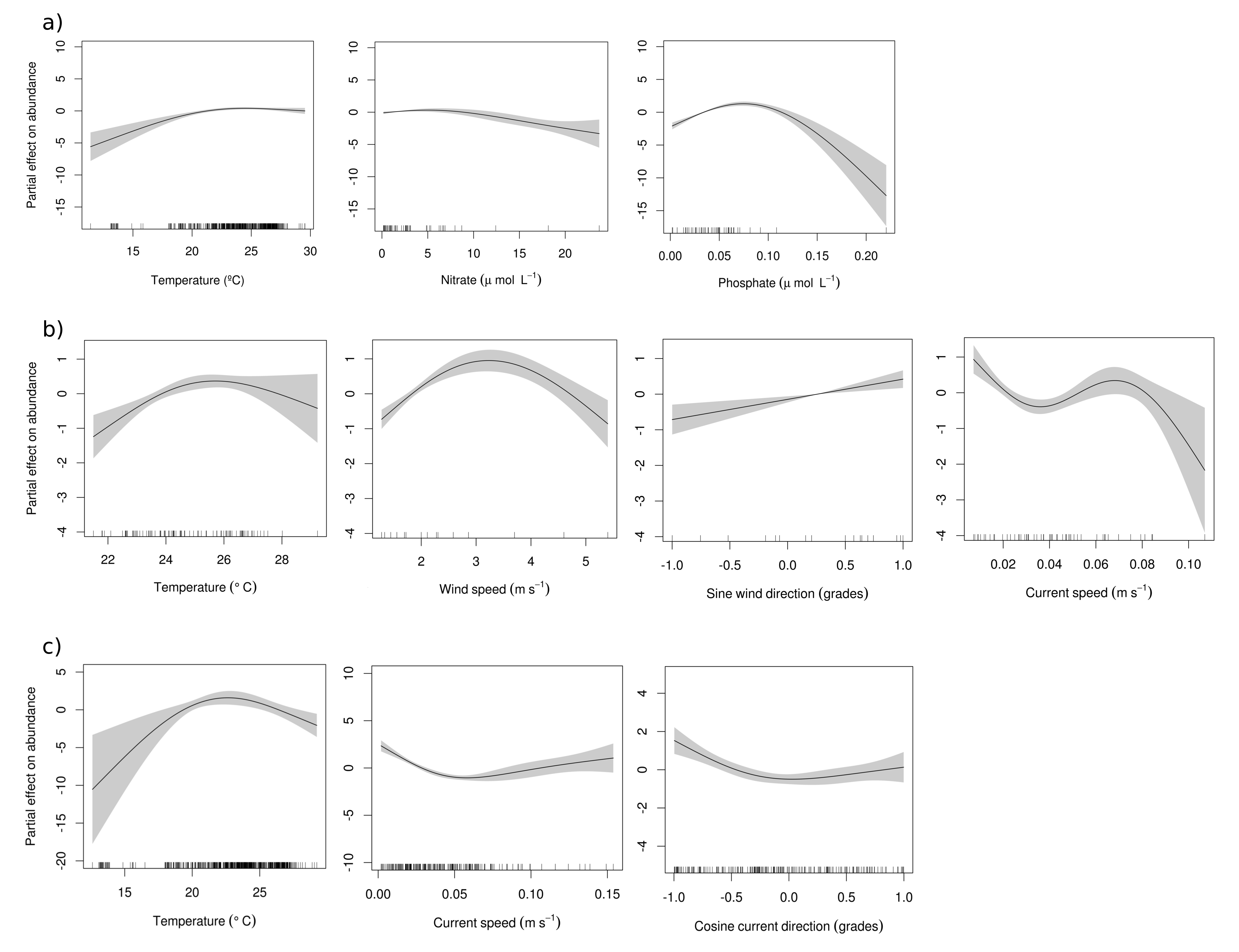

Supplement: S2 Fig — Central (bold) line show the best fit and the shaded areas show the 95% confidence intervals of the GAM-NB model. Bottom vertical lines represent observations. (TIFF) [file pone.0181611.s002.tiff]
