# Supplementary material for: Environmental factors influencing the spatio-temporal distribution of Carybdea marsupialis (Lineo, 1978, Cubozoa) in South-Western Mediterranean coasts
Source: PLoS One. 2017 Jul 26;12(7):e0181611. doi: 10.1371/journal.pone.0181611 (PMC5528890; doi:10.1371/journal.pone.0181611)
Supplement: S1 Table — (DOC) [file pone.0181611.s003.doc]

| MODEL | Mean | Variance | Model | Error Distribution |
| --- | --- | --- | --- | --- |
| GAM-P | $E\left(\Upsilon_{i}\right)=\mu_{i}$ | $var\left(\Upsilon_{i}\right)=\mu_{i}$ | $\Upsilon_{i}=\alpha+f\left(X_{ij}\right)+\varepsilon_{i}$ | $\varepsilon_{i}\text{\textasciitilde}N\left(\text{0,}\sigma^{2}\right)$ |
| GAM-NB | $E\left(\Upsilon_{i}\right)=\mu_{i}$ | $var\left(\Upsilon_{i}\right)=\mu_{i}+\frac{\mu_{i}^{2}}{k}$ | $\Upsilon_{i}=\alpha+f\left(X_{ij}\right)+\varepsilon_{i}$ | $\varepsilon_{i}\text{\textasciitilde}N\left(\text{0,}\sigma^{2}\right)$ |
| ZI-P | $E\left(\Upsilon_{i}\right)=\mu_{i}\times\left(1-\Pi_{i}\right)$ | $var\left(\Upsilon_{i}\right)=\left(1-\Pi_{i}\right)\times\left(\mu_{i}+\Pi_{i}\times\mu_{i}^{2}\right)$ |  | $\varepsilon_{i}=\frac{\Upsilon_{i}-\left(1-\Pi_{i}\right)\times\mu_{i}}{\sqrt{var\left(\Upsilon_{i}\right)}}$ |
| ZI-NB | $E\left(\Upsilon_{i}\right)=\mu_{i}\times\left(1-\Pi_{i}\right)$ | $var\left(\Upsilon_{i}\right)=\left(1-\Pi_{i}\right)\times\left(\mu_{i}+\frac{\mu_{i}^{2}}{k}\right)+\mu_{i}^{2}\times\left(\Pi_{i}^{2}+\Pi_{i}\right)$ | $P\left(\Upsilon_{i}=0\right)=\Pi_{i}+\left(1-\Pi_{i}\right)\times\left(\frac{\kappa}{\mu_{i}+\kappa}\right)^{\kappa}$ | $\varepsilon_{i}=\frac{\Upsilon_{i}-\left(1-\Pi_{i}\right)\times\mu_{i}}{\sqrt{var\left(\Upsilon_{i}\right)}}$ |
