# Supplementary material for: Environmental factors influencing the spatio-temporal distribution of Carybdea marsupialis (Lineo, 1978, Cubozoa) in South-Western Mediterranean coasts
Source: PLoS One. 2017 Jul 26;12(7):e0181611. doi: 10.1371/journal.pone.0181611 (PMC5528890; doi:10.1371/journal.pone.0181611)
Supplement: S2 Table — Minimum 'Min', maximum 'Max', sum 'Sum', mean 'Mean' and standard deviation 'SD' values are showed for each class size and sampling site. (DOC) [file pone.0181611.s004.doc]

| Site | Small | | | | | Medium | | | | | Large | | | | |
| --- | --- | --- | --- | --- | --- | --- | --- | --- | --- | --- | --- | --- | --- | --- | --- |
|  | Min | Max | Sum | Mean | SD | Min | Max | Sum | Mean | SD | Min | Max | Sum | Mean | SD |
| AL | 0.0  (0.0) | 117  (91.6) | 452  (269.7) | 4.5  (2.7) | 14.2  (10.6) | 1  (0.1) | 39  (6.6) | 147  (34.1) | 4.0  (0.9) | 6.5  (1.6) | 0.0  (0.0) | 53  (5.2) | 234  (18.6) | 2.2  (0.2) | 7.1  (0.6) |
| MO | 0.0  (0.0) | 324  (62.3) | 426  (111.2) | 4.7  (1.2) | 34.2  (6.7) | 1  (0.1) | 2  (0.3) | 6  (0.9) | 1.2  (0.2) | 0.5  (0.1) | 0.0  (0.0) | 4  (0.8) | 11  (1.5) | 0.1  (0.01) | 0.5  (0.1) |
| BB | 0.0  (0.0) | 80  (68.5) | 569  (418.3) | 5.4  (4.0) | 12.7  (9.6) | 1  (0.04) | 18  (1.9) | 75  (10.3) | 3.0  (0.4) | 3.8  (0.5) | 0.0  (0.0) | 5  (0.5) | 13  (1.3) | 0.1  (0.01) | 0.6  (0.1) |
| RA | 0.0  (0.0) | 119  (40.2) | 459  (184.8) | 5.2  (2.1) | 16.8  (5.8) | 1  (0.04) | 16  (1.6) | 51  (7.9) | 2.7  (0.4) | 4.2  (0.4) | 0.0  (0.0) | 4  (0.4) | 11  (1.2) | 0.1  (0.01) | 0.5  (0.1) |
| MC | 0.0  (0.0) | 61  (11.5) | 89  (24.7) | 1.2  (0.3) | 7.2  (1.5) | 1  (0.1) | 121  (16.6) | 134  (19.1) | 16.8  (2.4) | 42.2  (5.6) | 0.0  (0.0) | 4  (0.5) | 16  (2.0) | 0.2  (0.02) | 0.6  (0.1) |
| RO | 0.0  (0.0) | 2  (0.9) | 7  (3.4) | 0.1  (0.1) | 0.3  (0.2) | 1  (0.0) | 0  (0.0) | 0  (0.0) | 0.0  (0.0) | 0.0  (0.0) | 0.0  (0.0) | 0  (0.0) | 0  (0.0) | 0.0  (0.0) | 0.0  (0.0) |
| All  Sites | 0.0  (0.0) | 117  (91.6) | 2002  (1012.2) | 3.8  (1.9) | 18.0  (7.4) | 1  (0.0) | 121  (16.6) | 413  (72.3) | 4.4  (0.8) | 13.1  (2.0) | 0.0  (0.0) | 53  (5.2) | 285  (24.6) | 0.5  (0.04) | 3.1  (0.3) |
