# Supplementary material for: Environmental factors influencing the spatio-temporal distribution of Carybdea marsupialis (Lineo, 1978, Cubozoa) in South-Western Mediterranean coasts
Source: PLoS One. 2017 Jul 26;12(7):e0181611. doi: 10.1371/journal.pone.0181611 (PMC5528890; doi:10.1371/journal.pone.0181611)
Supplement: S3 Table — Values are shown by season. (DOC) [file pone.0181611.s005.doc]

| Variable | Site | Spring | | | | Summer | | | | Autumn | | | | Winter | | | |
| --- | --- | --- | --- | --- | --- | --- | --- | --- | --- | --- | --- | --- | --- | --- | --- | --- | --- |
|  |  | Min | Max | Mean | SD | Min | Max | Mean | SD | Min | Max | Mean | SD | Min | Max | Mean | SD |
| SST (ºC) | AL | 20.27 | 25.41 | 22.46 | 1.80 | 21.50 | 28.10 | 25.32 | 1.69 | 18.04 | 24.54 | 21.23 | 2.37 | 13.41 | 13.70 | 13.58 | 0.10 |
|  | MO | 20.15 | 25.16 | 22.24 | 1.62 | 20.71 | 27.14 | 25.20 | 1.57 | 18.17 | 24.10 | 20.69 | 2.33 | 13.21 | 13.37 | 13.29 | 0.06 |
|  | BB | 20.41 | 24.82 | 22.28 | 1.67 | 22.80 | 27.3 | 25.33 | 1.44 | 17.98 | 24.68 | 21.10 | 2.41 | 13.09 | 13.22 | 13.15 | 0.05 |
|  | RA | 21.19 | 27.1 | 23.45 | 2.23 | 23.28 | 29.54 | 25.97 | 1.88 | 18.23 | 24.49 | 21.17 | 2.31 | 13.20 | 13.36 | 13.27 | 0.06 |
|  | MC | 21.25 | 22.33 | 21.74 | 0.36 | 22.45 | 28.06 | 25.82 | 1.45 | 16.50 | 25.16 | 21.00 | 2.68 | 12.61 | 13.63 | 13.38 | 0.38 |
|  | RO | 19.56 | 23.79 | 21.56 | 1.63 | 22.28 | 27.86 | 25.27 | 1.57 | 14.87 | 24.37 | 20.64 | 3.19 | 11.42 | 13.88 | 13.51 | 0.79 |
|  | All Sites | 19.56 | 27.1 | 22.31 | 1.79 | 20.71 | 29.54 | 25.48 | 1.63 | 14.87 | 25.16 | 20.97 | 2.55 | 12.82 | 13.88 | 13.36 | 0.37 |
| Salinity | AL | 35.08 | 37.04 | 36.43 | 0.58 | 36.70 | 37.72 | 37.25 | 0.23 | 35.47 | 37.21 | 36.88 | 0.29 | 37.18 | 37.44 | 37.31 | 0.08 |
|  | MO | 35.70 | 37.07 | 36.93 | 0.53 | 36.03 | 37.76 | 37.28 | 0.27 | 36.19 | 37.33 | 36.92 | 0.19 | 36.40 | 37.40 | 37.19 | 0.33 |
|  | BB | 34.67 | 37.30 | 36.84 | 0.51 | 36.92 | 37.84 | 37.49 | 0.2 | 36.92 | 37.36 | 37.02 | 0.21 | 37.51 | 37.56 | 37.54 | 0.02 |
|  | RA | 32.98 | 37.39 | 36.91 | 0.87 | 36.02 | 37.83 | 37.48 | 0.25 | 36.38 | 37.41 | 37.01 | 0.29 | 35.63 | 37.64 | 37.22 | 0.77 |
|  | MC | 36.37 | 37.09 | 36.93 | 0.2 | 33.29 | 37.68 | 37.06 | 0.69 | 36.03 | 37.23 | 36.70 | 0.29 | 37.12 | 37.63 | 37.48 | 0.18 |
|  | RO | 35.63 | 37.22 | 36.82 | 0.33 | 36.72 | 37.76 | 37.40 | 0.21 | 35.78 | 37.62 | 37.02 | 0.37 | 37.07 | 37.57 | 37.47 | 0.15 |
|  | All Sites | 32.98 | 37.39 | 36.74 | 0.58 | 33.29 | 37.84 | 37.33 | 0.38 | 35.47 | 37.62 | 36.92 | 0.29 | 35.63 | 37.64 | 37.36 | 0.37 |
| Chl a (µg L^-1^) | AL | 0.18 | 0.30 | 0.26 | 0.06 | 0.55 | 2.03 | 1.18 | 0.62 | 0.64 | 0.96 | 0.77 | 0.13 | 0.50 | 0.50 | 0.50 | 0.00 |
|  | MO | 0.13 | 0.34 | 0.28 | 0.10 | 0.41 | 1.92 | 1.23 | 0.58 | 0.85 | 1.01 | 0.93 | 0.07 | 0.40 | 0.40 | 0.40 | 0.00 |
|  | BB | 0.10 | 0.15 | 0.14 | 0.03 | 0.36 | 2.00 | 0.98 | 0.63 | 0.52 | 1.03 | 0.68 | 0.23 | 0.25 | 0.25 | 0.25 | 0.00 |
|  | RA | 0.07 | 0.11 | 0.10 | 0.02 | 0.35 | 0.57 | 0.49 | 0.08 | 0.48 | 0.91 | 0.66 | 0.19 | 0.42 | 0.42 | 0.42 | 0.00 |
|  | MC | 0.11 | 0.11 | 0.11 | 0.00 | 0.17 | 0.40 | 0.28 | 0.08 | 0.17 | 0.34 | 0.24 | 0.07 | 0.16 | 0.16 | 0.16 | 0.00 |
|  | RO | 0.04 | 0.34 | 0.13 | 0.14 | 0.25 | 0.57 | 0.36 | 0.13 | 0.31 | 0.38 | 0.34 | 0.03 | 0.12 | 0.12 | 0.12 | 0.00 |
|  | All Sites | 0.04 | 0.34 | 0.17 | 0.11 | 0.17 | 2.03 | 0.76 | 0.59 | 0.17 | 1.03 | 0.60 | 0.28 | 0.12 | 0.50 | 0.32 | 0.00 |
| Nitrate (µmol L^-1^) | AL | 1.53 | 2.14 | 1.74 | 0.30 | 1.09 | 2.79 | 1.99 | 0.74 | 2.63 | 6.24 | 4.51 | 1.57 | 2.75 | 2.75 | 2.75 | 0.00 |
|  | MO | 2.64 | 18.15 | 13.38 | 7.30 | 1.00 | 5.33 | 2.62 | 1.73 | 2.64 | 23.71 | 10.28 | 8.92 | 12.42 | 12.42 | 12.42 | 0.00 |
|  | BB | 0.73 | 3.13 | 2.39 | 1.13 | 0.55 | 6.89 | 2.91 | 2.58 | 2.61 | 7.97 | 5.09 | 2.19 | 6.63 | 6.63 | 6.63 | 0.00 |
|  | RA | 0.22 | 0.22 | 0.22 | 0.00 | 0.22 | 0.93 | 0.47 | 0.28 | 0.93 | 3.07 | 2.17 | 0.94 | 6.41 | 6.41 | 6.41 | 0.00 |
|  | MC | 0.89 | 0.89 | 0.89 | 0.00 | 0.18 | 0.6 | 0.31 | 0.17 | 0.27 | 8.71 | 3.85 | 3.63 | 2.73 | 2.73 | 2.73 | 0.00 |
|  | RO | 0.44 | 4.92 | 3.54 | 2.11 | 0.21 | 2.95 | 1.12 | 1.10 | 0.93 | 1.70 | 1.35 | 0.34 | 0.83 | 0.84 | 0.84 | 0.00 |
|  | All Sites | 0.22 | 18.15 | 4.03 | 5.64 | 0.18 | 6.89 | 1.58 | 1.71 | 0.27 | 23.71 | 4.61 | 5.04 | 0.83 | 12.42 | 5.45 | 3.89 |
| Phosphate (µmol L^-1^) | AL | 0.06 | 0.07 | 0.06 | 0.00 | 0.03 | 0.11 | 0.06 | 0.03 | 0.03 | 0.05 | 0.03 | 0.01 | 0.03 | 0.03 | 0.03 | 0.00 |
|  | MO | 0.04 | 0.07 | 0.05 | 0.01 | 0.03 | 0.06 | 0.05 | 0.02 | 0.03 | 0.07 | 0.06 | 0.02 | 0.02 | 0.02 | 0.02 | 0.00 |
|  | BB | 0.03 | 0.06 | 0.04 | 0.01 | 0.04 | 0.06 | 0.05 | 0.01 | 0.02 | 0.05 | 0.04 | 0.01 | 0.04 | 0.04 | 0.04 | 0.00 |
|  | RA | 0.03 | 0.06 | 0.04 | 0.01 | 0.02 | 0.05 | 0.03 | 0.01 | 0.03 | 0.22 | 0.11 | 0.08 | 0.05 | 0.05 | 0.05 | 0.00 |
|  | MC | 0.05 | 0.05 | 0.05 | 0.00 | 0.01 | 0.05 | 0.02 | 0.02 | 0.00 | 0.04 | 0.02 | 0.02 | 0.02 | 0.02 | 002 | 0.00 |
|  | RO | 0.02 | 0.07 | 0.03 | 0.02 | 0.02 | 0.06 | 0.04 | 0.02 | 0.00 | 0.06 | 0.04 | 0.02 | 0.08 | 0.08 | 0.08 | 0.00 |
|  | All Sites | 0.02 | 0.07 | 0.05 | 0.01 | 0.02 | 0.11 | 0.04 | 0.02 | 0.00 | 0.22 | 0.05 | 0.05 | 0.02 | 0.08 | 0.04 | 0.02 |
| SPM (mg L^-1^) | AL | 16.12 | 24.92 | 21.87 | 4.27 | 10.64 | 24.71 | 16.69 | 5.20 | 10.64 | 26.32 | 18.27 | 6.45 | 12.83 | 12.83 | 12.83 | 0.00 |
|  | MO | 17.39 | 28.69 | 25.21 | 5.32 | 10.83 | 17.74 | 14.95 | 2.92 | 13.38 | 33.94 | 23.00 | 8.32 | 11.52 | 11.53 | 11.53 | 0.00 |
|  | BB | 16.25 | 25.03 | 22.32 | 4.13 | 11.87 | 24.22 | 15.79 | 4.94 | 12.45 | 22.45 | 16.95 | 4.10 | 16.59 | 16.59 | 16.59 | 0.00 |
|  | RA | 10.59 | 19.86 | 16.64 | 4.52 | 12.36 | 27.92 | 17.39 | 6.17 | 13.86 | 22.85 | 17.99 | 3.82 | 18.79 | 18.80 | 18.80 | 0.00 |
|  | MC | 12.09 | 12.09 | 12.09 | 0.00 | 8.11 | 14.70 | 14.24 | 2.36 | 10.98 | 16.00 | 14.24 | 2.27 | 17.30 | 17.30 | 17.30 | 0.00 |
|  | RO | 13.00 | 16.51 | 14.08 | 1.65 | 7.65 | 15.02 | 11.59 | 2.80 | 9.50 | 13.08 | 11.20 | 1.56 | 11.44 | 11.44 | 11.44 | 0.00 |
|  | All Sites | 10.59 | 28.69 | 19.36 | 5.98 | 7.65 | 27.92 | 14.67 | 4.89 | 9.50 | 33.94 | 17.00 | 6.16 | 11.44 | 18.80 | 14.60 | 2.97 |
| Current speed (m s^-1^) | AL | 0.02 | 0.03 | 0.03 | 0.00 | 0.01 | 0.08 | 0.05 | 0.02 | 0.01 | 0.06 | 0.03 | 0.02 | 0.01 | 0.01 | 0.01 | 0.00 |
|  | MO | 0.03 | 0.15 | 0.08 | 0.05 | 0.03 | 0.15 | 0.08 | 0.03 | 0.01 | 0.07 | 0.03 | 0.02 | 0.05 | 0.05 | 0.05 | 0.00 |
|  | BB | 0.03 | 0.04 | 0.04 | 0.00 | 0.03 | 0.09 | 0.06 | 0.02 | 0.01 | 0.07 | 0.03 | 0.02 | 0.04 | 0.04 | 0.04 | 0.00 |
|  | RA | 0.02 | 0.04 | 0.03 | 0.01 | 0.01 | 0.09 | 0.04 | 0.02 | 0.02 | 0.05 | 0.04 | 0.01 | 0.04 | 0.04 | 0.04 | 0.00 |
|  | MC | 0.01 | 0.02 | 0.01 | 0.01 | 0.01 | 0.14 | 0.03 | 0.03 | 0.00 | 0.03 | 0.02 | 0.01 | 0.02 | 0.02 | 0.02 | 0.00 |
|  | RO | 0.01 | 0.06 | 0.03 | 0.02 | 0.00 | 0.12 | 0.05 | 0.03 | 0.01 | 0.06 | 0.04 | 0.02 | 0.07 | 0.07 | 0.07 | 0.00 |
|  | All Sites | 0.01 | 0.15 | 0.04 | 0.03 | 0.00 | 0.15 | 0.05 | 0.03 | 0.00 | 0.07 | 0.03 | 0.02 | 0.01 | 0.07 | 0.04 | 0.00 |
| Wind speed (m s^-1^) | All Sites | 2.00 | 4.60 | 3.32 | 1.04 | 1.28 | 5.40 | 2.67 | 1.39 | 0.75 | 2.00 | 1.39 | 0.42 | 1.03 | 1.03 | 1.03 | 0.00 |
