# Supplementary material for: Environmental factors influencing the spatio-temporal distribution of Carybdea marsupialis (Lineo, 1978, Cubozoa) in South-Western Mediterranean coasts
Source: PLoS One. 2017 Jul 26;12(7):e0181611. doi: 10.1371/journal.pone.0181611 (PMC5528890; doi:10.1371/journal.pone.0181611)
Supplement: S4 Table — The Pearson correlation coefficient (r), Spearman rank correlation (p), intercept (α) and slope (b) of a linear regression between the observed versus fitted values, Akaike Information Criterion (AIC), log likelihood (Log lik) and degrees of freedom (Df) are showed for each size class of Carybdea marsupialis medusae. NA = Not Available. (DOC) [file pone.0181611.s006.doc]

|  | Small | | | | | | | Medium | | | | | | | Large | | | | | | |
| --- | --- | --- | --- | --- | --- | --- | --- | --- | --- | --- | --- | --- | --- | --- | --- | --- | --- | --- | --- | --- | --- |
| Model | r | p | α | b | AIC | Log lik | Df | r | p | α | b | AIC | Log lik | Df | r | p | α | b | AIC | Log lik | Df |
| GAM-P | 0.51 | 0.64 | 2.85 | 0.28 | 4371.57 | -2155.90 | 30 | 0.60 | 0.31 | 2.90 | 0.30 | 765.06 | -356.80 | 26 | 0.89 | 0.41 | 0.09 | 0.80 | 522.20 | -235.90 | 25 |
| GAM-NB | 0.44 | 0.68 | 3.04 | 0.23 | 1818.62 | -883.20 | 26 | 0.47 | 0.31 | 3.50 | 0.16 | 478.89 | -220.20 | 19 | 0.84 | 0.43 | 0.10 | 0.80 | 462.70 | -205.40 | 26 |
| ZI-P | 0.14 | 0.24 | 4.60 | 0.05 | 5475.66 | -2714.00 | 24 | NA | NA | NA | NA | NA | NA | NA | 0.12 | 0.10 | 0.90 | 0.10 | 632.30 | -292.20 | 24 |
| ZI-NB | 0.06 | 0.50 | 6.50 | 0.07 | 1646.90 | -798.50 | 25 | NA | NA | NA | NA | NA | NA | NA | 0.40 | 0.40 | 0.40 | 0.30 | 490.40 | -220.20 | 25 |
